# Recurring Amplification at 11q22.1-q22.2 Locus Plays an Important Role in Lymph Node Metastasis and Radioresistance in OSCC

**DOI:** 10.1038/s41598-017-16247-y

**Published:** 2017-11-22

**Authors:** Priyanka G. Bhosale, Manishkumar Pandey, Simona Cristea, Mickey Shah, Asawari Patil, Niko Beerenwinkel, Alejandro A. Schäffer, Manoj B. Mahimkar

**Affiliations:** 1 Cancer Research Institute (CRI), Advanced Centre for Treatment, Research and Education in Cancer (ACTREC), Tata Memorial Centre (TMC), Navi Mumbai, 410210 India; 2 Homi Bhabha National Institute, Training School Complex, Anushakti Nagar, Mumbai, 400085 India; 3 Department of Biosystems Science and Engineering, ETH Zurich, 4058 Basel, Switzerland; 4 SIB Swiss Institute of Bioinformatics, 4058 Basel, Switzerland; 5 Department of Biostatistics and Computational Biology, Dana-Farber Cancer Institute, Boston, Massachusetts 02115 USA; 6 Department of Biostatistics, Harvard T.H. Chan School of Public Health, Boston, Massachusetts 02115 USA; 7 Department of Pathology, Tata Memorial Hospital, Tata Memorial Centre (TMC), Parel, Mumbai, 400012 India; 8 Computational Biology Branch, National Center for Biotechnology Information, National Institute of Health (NIH), Department of Health and Human Services (DHHS), Bethesda, Maryland 20894 USA

## Abstract

A key feature in the pathogenesis of OSCC is genetic instability, which results in altered expression of genes located in amplified/deleted chromosomal regions. In a previous study we have shown that the amplification of the 11q22.1-q22.2 region, encoding cIAP1 and cIAP2, is associated with lymph node metastasis and poor clinical outcome in OSCC. Here, we validate the aCGH results by nuc ish and detect a weak amplification at the 11q22.1-q22.2 locus in 37% of the 182 samples tested. We find positive correlation of 11q22.1-q22.2 amplification with lymph node metastasis, reduced survival, and increased cancer recurrence, and we observe that patients with 11q22.1-q22.2 amplification fail to respond to radiotherapy. We confirm the concurrent overexpression of cIAP1 and cIAP2 and observe differential subcellular localization of the two proteins in OSCC. To ascertain the roles of cIAP1/cIAP2 in lymph node metastasis and radioresistance, we use an *in vitro* pre-clinical model and confirm the role of cIAP1 in invasion and the role of cIAP2 in invasion and migration. Studies of other tumor types in which cIAP1 is overexpressed suggest that multi-regimen treatments including SMAC mimetics may be effective. Thus, the evaluation of 11q22.1-q22.2 amplifications in OSCC patients may help choose the most effective treatment.

## Introduction

Lymph node metastasis, tumor stage, and tumor recurrence are important prognostic factors in oral cancer patients. Most oral squamous cell carcinoma (OSCC) patients succumb either to overtreatment, *i*.*e*. physical morbidity due to elective neck dissection, or to side effects of post-operative radiochemotherapy^1–3^. An important risk factor for OSCC is infection by human papilloma virus (HPV). HPV-positive and HPV-negative OSSCs have different genomic characteristics^4,5^. We have previously identified copy number alterations (CNAs) associated with lymph node metastasis and clinical outcome in HPV-negative OSCC patients^6^. We found that the amplification of the 11q22.1-q22.2 chromosomal region is associated with poor clinical outcome, and the amplification of 11q13.3 strongly correlates with disease progression^6,7^. A study of The Cancer Genome Atlas (TCGA) data on head and neck squamous cellular carcinoma (HNSCC) also highlights the co-amplification of 11q13 (including the genes *CCND1*, *FADD* and *CTTN*) and 11q22 (including the genes *BIRC2* and *YAP1*) as a common occurrence in HPV-negative HNSCC^8^. Moreover, the International Cancer Genome Consortium (ICGC) reports the up-regulation of genes located on the 11q13 and 11q22 loci in more than 10% of OSCC patients^9^, indicating important roles for the 11q22 and 11q13 amplifications in oral tumorigenesis.

Together with the 11q13 amplification in OSCC, it has also been reported that the genes *ORAOV1*, *ANO1*, *CCND1*, *FGF3*, *FGF4*, and *FADD* in the 11q13 region are overexpressed, indicating that they are likely oncogenic drivers with important roles in metastasis that provide cancer cells with a selective advantage^10^. The 11q22.1–22.2 locus includes two genes, *BIRC2* (cIAP1) and *BIRC3* (cIAP2), that code for cellular Inhibitor of Apoptosis Proteins (cIAPs), which are known to promote cell survival in tumors through regulation of apoptosis^11^. Higher expression of either cIAP1 or cIAP2 has been reported in therapy resistant glioblastoma, cervical cancer, and OSCC^12–14^. In addition, cIAP1 has been shown to potentially be involved in the progression or metastasis formation of non-small cell lung cancer, cervical cancer, gastrointestinal stromal tumors (GIST), tongue cancer, HNSCC, and acute myeloid leukemia^13,15–19^. Since *BIRC2* and *BIRC3* are adjacent, paralogous genes on human chromosome 11, it is not surprising that overexpression of cIAP2 has also been associated with the progression of the same tumor types and with treatment resistance^12,19–22^. The mechanism by which cIAP1/cIAP2 overexpression promotes oncogenesis is by regulating TNFα-mediated activation of canonical NFκB signaling, while suppressing the alternative NFκB pathway^23–25^. The signaling is cyclic, such that expression of these proteins is also regulated by NFκB^26^. One of the downstream effects of NFκB activation by cIAP1/cIAP2 is to promote transcription of beclin 1 and thereby to promote autophagy, which enhances cell survival^27^, although a different study suggested that under some conditions autophagy could lead to degradation of cIAP1/cIAP2 and cell death^28^.

In contrast to overexpression in some tumor types, *BIRC3* is sometimes deleted or mutated in chronic lymphocytic leukemia (CLL) and other lymphoid malignancies, and alterations in *BIRC3* have also been associated with treatment resistance^29–35^. Furthermore, somatic mutations in both genes that appear to inactivate the NFκB signaling function of either cIAP1 or cIAP2 have been reported in several solid tumors^36^. One type of blood cancer that has recurrent amplifications and overexpression of cIAP1/cIAP2 is the activated B cell (ABC) subtype of diffuse B cell lymphomas^37^.

The roles of cIAP1 and cIAP2 in lymph node metastasis and therapy resistance in OSCC have not been thoroughly investigated. Understanding these roles is important because these proteins can both be targeted by drugs called SMAC (second mitochondrial activator of caspase) mimetics^18,19,25,38^. SMAC, also known as DIABLO, promotes cell death by cleavage and inactivation of all IAP proteins. SMAC mimetics bind to IAP proteins in the same way that SMAC does, and have been shown to be part of effective single-agent or multi-agent treatment for HNSCC cell lines, GIST cell lines overexpressing cIAP1, a nasopharygeal cancel cell line overexpressing cIAP1, and ABC diffuse B cell lymphomas^19,38–40^. In a small-scale single-agent trial in ovarian cancer, a SMAC mimetic downregulated IAP proteins, but did not lead to clinical benefit^41^.

Here, we validate the amplifications of 11q13.3 and 11q22.1-q22.2 in OSCC and evaluated the expression of *BIRC2* and *BIRC3* with respect to lymph node metastasis and poor survival in oral cancer patients. The current study also delineates the association between the 11q22 amplification and radioresistance; cIAP1/2 expression levels emerge as independent predictors of patient survival. Based on functional validation, we demonstrate the roles of cIAP1 and cIAP2 in invasion or metastasis in OSCC. Our findings of resistance to radiation treatment in OSCC patients with 11q22 amplification, together with other studies showing effectiveness of SMAC mimetics to target cIAP1 overexpression, suggest that cIAP1 overexpression or 11q22 amplification could be used as biomarkers to guide personalized treatment for OSCC.

## Results

### Clinicopathological and demographic characteristics

The clinicopathological and demographic characteristics of all leukoplakia (OPL) and OSCC patients analyzed in this study are summarized in Table 1. In total, nuclear *in-situ* hybridization (nuc ish) and quantitative reverse transcriptase PCR (qRT-PCR) were performed on n = 182 and n = 135 OSCC samples, respectively, while immunohistochemistry (IHC) was performed on 57 leukoplakia and 132 OSCC samples. All the study samples are negative for high risk HPV^42^. Forty-eight tumor samples are overlapping with the previous aCGH study^6^, and the remaining samples formed the independent validation set. The patients included in this study were predominantly male (~80%), with a median age of ~45 years (inter quartile range of 39–58). The major proportion of study patients had gingivobuccal complex (GBC) cancers (~85%), and the remaining patients had tongue cancers (~15%). Almost equal proportions of samples were lymph node metastasis positive (48%) and negative (52%).

**Table 1:** The Demographic details of study samples.

**Patient characteristic**	**nuc ish n = 182** (**%**)	**qRT-PCR n = 154** (**%**)	**IHC n = 209** (**%**)
**Total study samples**			
OSCC	182	135	132
Leukoplakia (OPL)	NA	NA	57
Healthy Normal	NA	19	20
**Age at diagnosis**			
Median (IQR)	50 (42–58)	50 (42–59)	47 (39–57)
**Gender**			
Male	138 (75.8%)	101 (75%)	169 (81%)
Female	44 (24.2%)	34 (25%)	40 (19%)
**Site of OSCC**			
Gnigivobuccal complex	150 (82.4%)	135 (100%)	162 (85.7%)
Tongue	32 (17.6%)	0 (0%)	27 (14.3%)
**Pathological stage**			
Stage 1 and 2 (Early stage OSCC)	33 (18.1%)	56 (41.5%)	25 (18.9%)
Stage 3 and 4 (Advanced stage OSCC)	149 (81.9%)	79 (58.5%)	107 (81.1%)
**Pathological cervical lymph node involvement** (**N**)			
Node negative (N0)	94 (51.6%)	79 (58.5%)	79 (59.8%)
Node positive (N+)	88 (48.4%)	56 (41.5%)	53 (40.2%)
**Pathological grade**			
Well	11 (6.1%)	12 (8.9%)	9 (6.8%)
Moderate	126 (69.2%)	87 (64.4%)	82 (62.1%)
Poor	45 (24.7%)	36 (26.7%)	41 (31.1%)
**Treatment**			
Surgery	52 (28.6%)	60 (44.4%)	5 (3.8%)
Surgery + RT	78 (42.9%)	62 (46.0%)	79 (59.8%)
Surgery + CT	4 (2.2%)	1 (0.7%)	NA
Surgery + RT + CT	48 (26.3%)	12 (8.9%)	48 (36.4%)
**Radiation compliant cases**			
50–60 Gy	93 (51.1%)	63 (47%)	85 (64.4%)
<50 Gy	89 (48.9%)	72 (53%)	2 (1.5%)
No information	NA	NA	45 (34.1%)
**Habit profile**			
No Habit	3 (1.6%)	3 (2.2%)	8 (4.2%)
Exclusive tobacco users	63 (34.6%)	79 (58.5%)	66 (34.9%)
Exclusive smoker	3 (1.7%)	2 (1.5%)	16 (8.5%)
Mixed habit**	32 (17.6%)	28 (20.7%)	62 (32.8%)
No information	81 (44.5%)	23 (17%)	37 (19.6%)

NA: Not applicable; N: Tumor classification based on lymph node metastasis; IQR: Inter quartile range; RT: Radiotherapy; CT: Chemotherapy; Gy: Gray; **Mixed Habit: Tobacco chewing along with bidi/cigarette smoking and/or alcohol users.

### Recurrent copy number amplification of two 11q loci in OSCC

**Figure 1:**
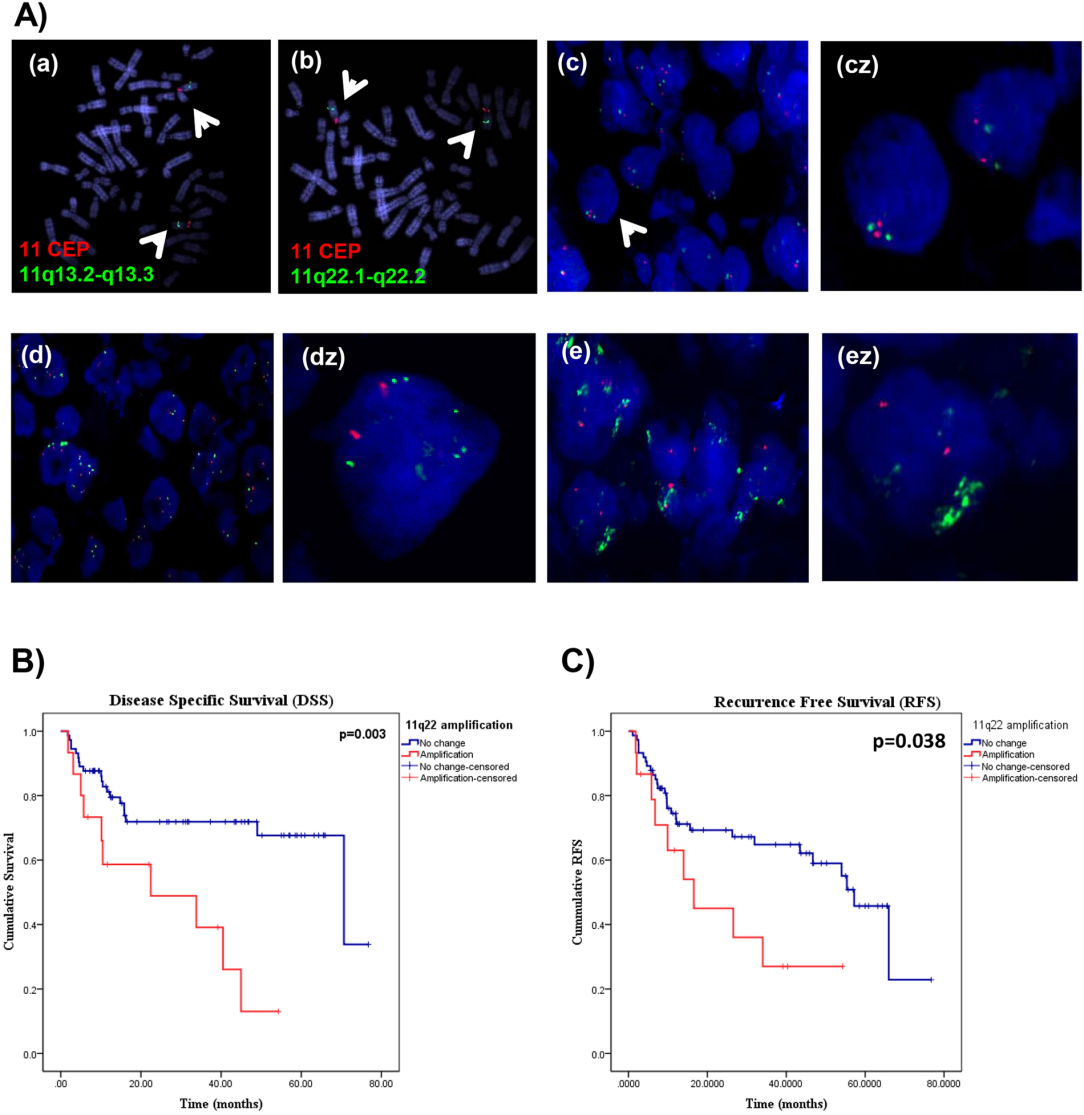
Copy number amplification of 11q13.3 and 11q22.1-q22.2 in OSCC. (**A**) nuc ish on metaphase chromosome confirmed the specificity of clones for 11CEP (red) and 11q locus (green) for (**a**) 11q13.3 (**b**) 11q22.1-q22.2. nuc ish signals were categorized as (**c**) normal (diploid nuclei), (**d**) weak amplification and (**e**) strong amplification, nuclei counterstained with DAPI (blue), original magnification 630X. Respective single nuclei zoom for each category are depicted in **cz**, **dz and ez**. (**B**) Kaplan-Meier plot representing disease specific survival (DSS) of patient groups without 11q22.1-q22.2 alteration and with 11q22.1-q22.2 strong amplification (p = 0.003). (**C**) Kaplan-Meier plot representing recurrence-free survival (RFS) of patient groups without 11q22.1-q22.2 alteration and with 11q22.1-q22.2 strong amplification (p = 0.038).

Our previous analysis of the aCGH data revealed multiple aberrations on chromosome 11, among which 11q13.3 and 11q22.1-q22.2 were significantly amplified and associated with disease advancement in HPV-negative gingivobuccal cancers^6,7^. In addition, our previous study showed that the 11q22.1 amplification was present in patients with lymph node metastasis and associated with poor survival in OSCC patients (Supplementary Figure S1). To validate these 11q alterations, we performed nuc ish analysis in 182 OSCC cases. The centromeric 11 CEP (Red) and the region-specific probes for 11q13.3 (Green), as well as for 11q22.1-q22.2 (Green), hybridized to their target loci and showed no cross reactivity (Fig. 1A (a,b)). Alterations for both 11q13.3 and 11q22.1-q22.2 were scored as either no change (i.e., diploid nuclei), weak amplification, or strong amplification (Fig. 1A, (c,d,e) with respective zoom (z)). 11q13.3 and 11q22.1-q22.2 alterations were observed in 53% and 37.5% OSCC samples respectively (Supplementary Table S1). We observed a positive correlation between a strong amplification in 11q13.3 and disease advancement (Supplementary Table S2), which is consistent with our previous aCGH results^6^. Both 11q13.3 and 11q22.1-q22.2 strong amplifications demonstrated significant correlation with lymph nodal metastasis (Supplementary Table S2). Using binomial logistic regression, we found a strong effect of the 11q22.1-q22.2 amplification on node metastasis, irrespective of the 11q13.3 alteration status (Table 2).

**Table 2:** Correlation of 11q13.3 and 11q22.1-q22.2 alterations with nodal metastasis.

Variable	Total cases	N+ cases (%)	OR (95% CI)	p* value
11q22 & 11q13 both change^‡^	88	36 (40.9)	**7**.**05** (2.72–18.29)	<0.0001
11q22 change & no 11q13 change	63	22 (34.92)	**7**.**23** (1.67–31.21)	0.008
11q13 change & no 11q22 change	78	27 (34.61)	2.71 (1.01–7.25)	0.047

^*^Binary logistic regression analysis; ^‡^Change: Includes both weak and strong amplification; OR: Odds Ratio; N+: Node metastasis positive; 11q13:11q13.3; 11q22: 11q22.1-q22.2.

### Association between 11q alterations and patient survival and tumor recurrence

The strong amplification of 11q22.1-q22.2 was found to be a predictor of poor clinical outcome in terms of recurrence (*p* = 0.043) and survival (*p* = 0.004). Specifically, the associated risk was higher in radiation-compliant patients (patients who have completed > 50 Gy radiation treatment) (Table 3A). Kaplan-Meier survival curves for the 11q22.1-q22.2 alterations are shown in Fig. 1B and C. Multivariate Cox proportional hazards models were used to assess the effect of multiple parameters on survival, highlighting that, in addition to node metastasis, the 11q22.1-q22.2 strong amplification is an independent predictor of poor patient survival (Table 3B).

**Table 3:** HR: Hazard Ratio;

A: Association of 11q22.1-q22.2 strong amplification with DSS and RFS						
	Disease specific survival			Recurrence free survival		
	Total/Death (%)	HR (95% CI)	p* value	Total/Relapse (%)	HR (95% CI)	p* value
Total cohort	88/30 (34.0%)	1.75 (1.19–2.57)	**0.004**	89/38 (42.6%)	1.483 (1.01–2.17)	0.043
Radiation compliant patients	55/16 (29.0%)	2.25 (1.29–3.91)	**0.004**	56/24 (42.8%)	2.25 2.15 (1.31–3.53)	0.002
**B: The association of multiple parameters on clinical outcome**						
**Clinicopathological parameter**	**Disease specific survival**					
	**HR** (**95% CI**)	***p*** ** value				
Nodal status	3.897 (1.03–14.73)	**0**.**045**				
Tumor stage	0.671 (0.12–3.49)	0.636				
11q13.3 strong amplification	0.460 (0.08–2.59)	0.379				
11q22.1-q22.2 strong amplification	7.126 (1.21–41.71)	**0**.**029**				

**Multivariate Cox Regression Analysis. HR: Hazard Ratio; *Cox Regression analysis.

### *BIRC2* and *BIRC3*: targets on 11q22.1-q22.2 amplicon

The 11q22.1-q22.2 amplicon includes a cluster of matrix metalloproteinase (*MMP*) genes and two members of the BIRC family, namely *BIRC2* and *BIRC3*, encoding cIAP1 and cIAP2, respectively (Supplementary Figure S1C). The upregulation of *MMP3*, *MMP7*, *MMP9* and their association with metastasis have been reported in various cancers^7,43,44^, and our previous analyses reported increased expression of *MMP3* in OSCC samples^6,45,46^. Here, we validated this finding using semi-quantitative RT-PCR and IHC (Supplementary Figure S2). Although we observed a significant increase in *MMP3* expression in OSCC samples compared to normal tissues, no significant association was observed between *MMP3* expression and lymph node metastasis or patient survival (data not shown).

**Figure 2:**
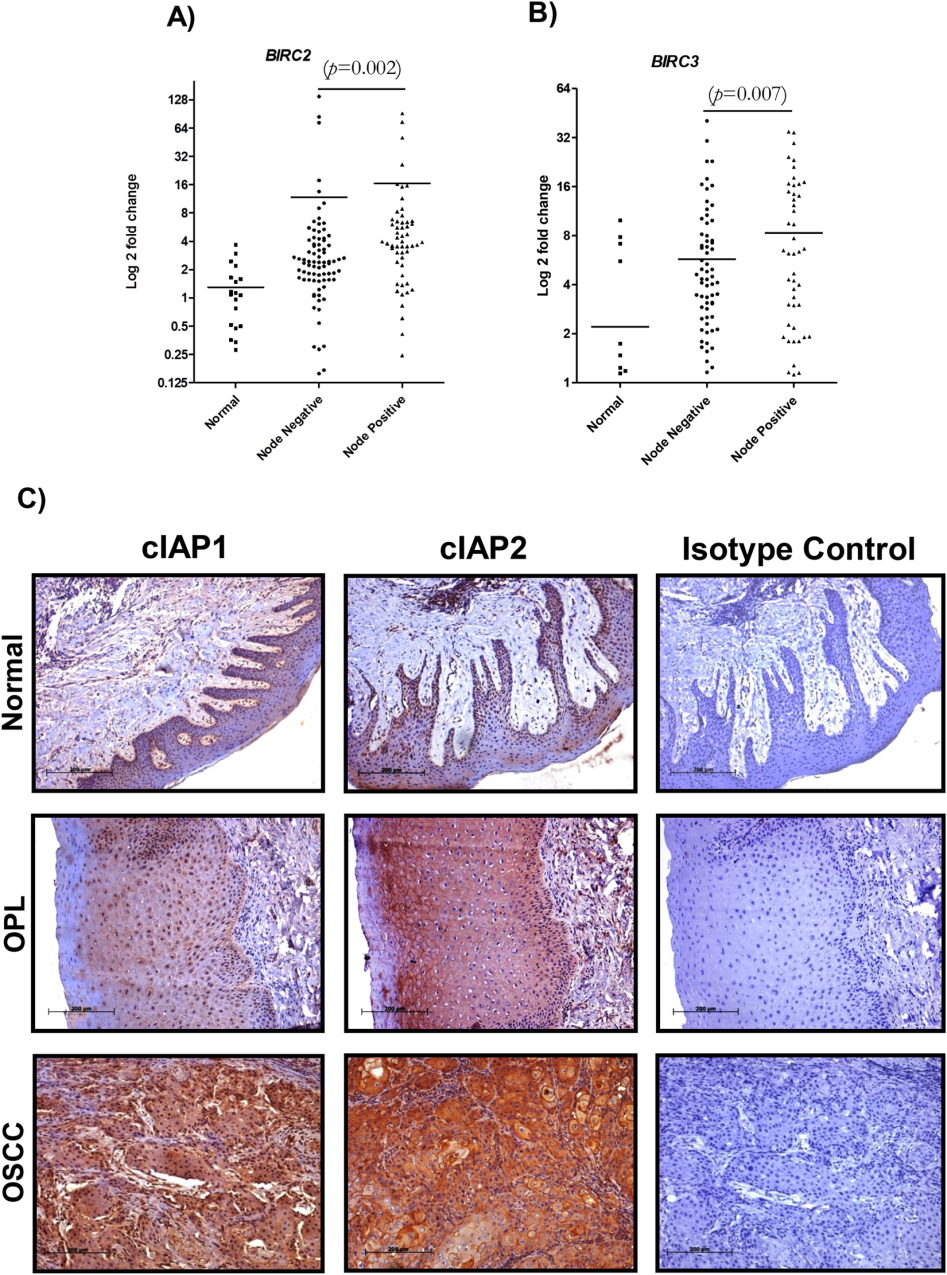
Correlation of *BIRC2* and *BIRC3* expression with OSCC progression and lymph node metastasis. (**A**,**B**) Upregulation of *BIRC2* and *BIRC3* in patients with lymph node metastasis, as compared to controls and patients negative for node metastasis. *p* represents the p-value. (**C**) IHC staining of cIAP1 and cIAP2 in representative normal buccal mucosa, leukoplakia (OPL), and OSCC tissues, shown along with the respective isotype control at 100X original magnification.

qRT-PCR based analysis revealed a significant increase in the expression of *BIRC2* and *BIRC3* in OSCCs as compared to normal tissue, and was associated with an increased risk of OSCC development (*BIRC2*: p = 0.001, OR = 0.133, 95% CI = 0.042–0.425; *BIRC3*: p = 0.002, OR = 0.166, 95% CI = 0.052–0.528). Upregulation of both *BIRC2* and *BIRC3* was significantly associated with lymph node metastasis (Fig. 2A and B, p < 0.002 and p < 0.007, respectively). In addition, Kaplan-Meier survival analysis demonstrated that the upregulation of either *BIRC2* or *BIRC3* predicts poor clinical outcome in OSCC patients (Supplementary Figure S3).

**Figure 3:**
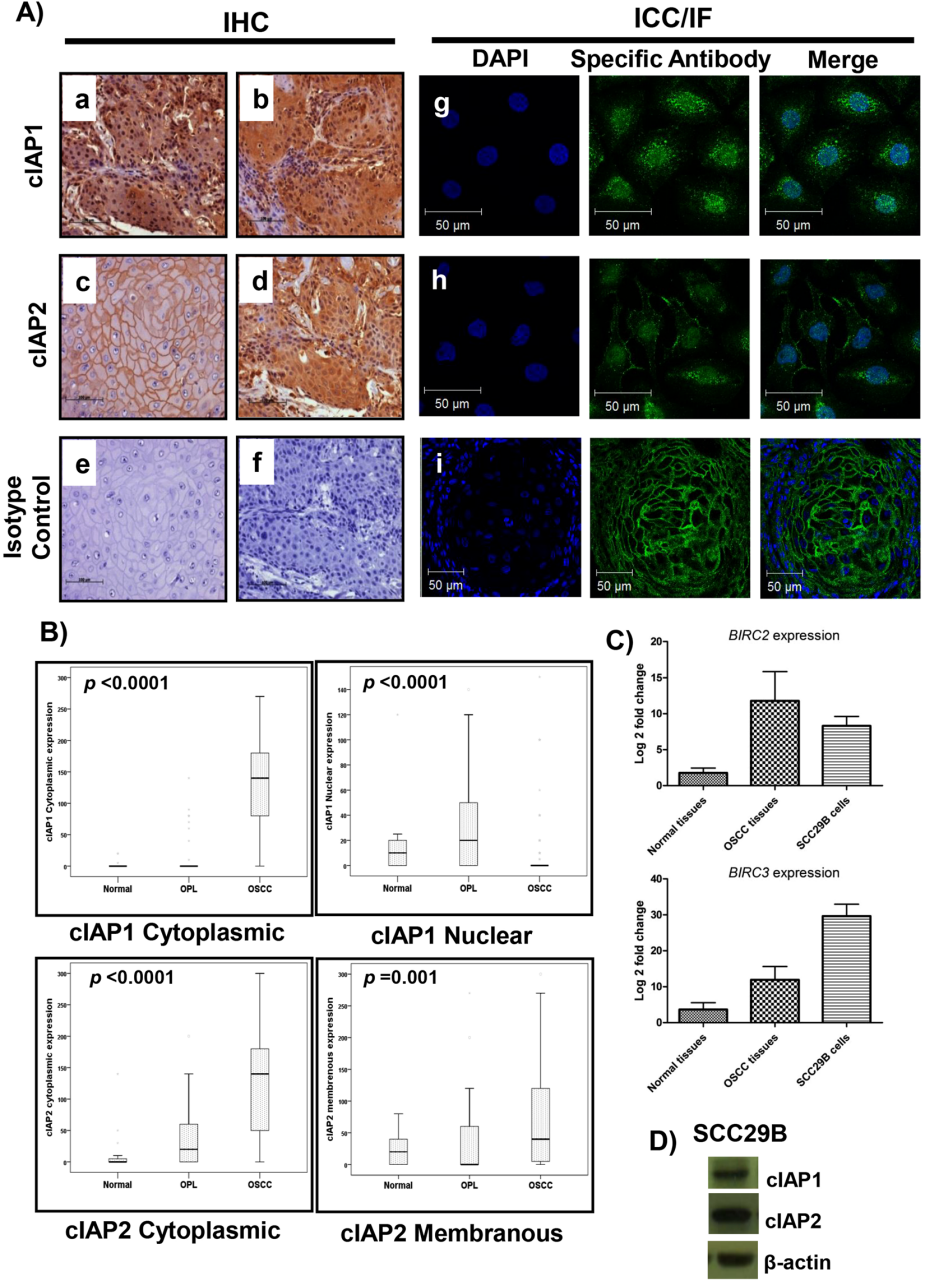
Differential subcellular localization of cIAP1 and cIAP2 and their overexpression in OSCC. (**A**) IHC staining (original magnification 100X) shows (**a**,**b**) nuclear or cytoplasmic expression of cIAP1 and (**c**,**d**) membranous or cytoplasmic expression of cIAP2 in OSCC. Respective isotype controls are represented in **e** and **f**. (**i**) Localization of cIAP2 at the cell membrane was confirmed by immunofluorescent (IF) staining in OSCC tumors. Nuclear/cytoplasmic localization of (**g**) cIAP1 and (**h**) membranous and cytoplasmic staining of cIAP2 were confirmed by ICC in SCC29B cells. For IF (original magnification 400X) and ICC (original magnification 630X), cells were stained with the nuclear stain DAPI (blue), as well as cIAP1/cIAP2 primary antibodies and Alexafluor 488 secondary antibodies (green) along with merge image. (**B**) Significant increase in cytoplasmic cIAP1 and cIAP2 in OSCC, as compared to normal and leukoplakia. Nuclear expression of cIAP1 and membranous expression of cIAP2 were more prominent in normal and leukoplakia (OPL) than in OSCC. y-axis represents the H-score for cIAP1/2 expression. (**C**) Comparable expression of *BIRC2* and *BIRC3* in SCC29B and OSCC tissues, with respect to normal gingivobuccal tissue, evaluated by qRT-PCR. (**D**) Expression of cIAP1 and cIAP2 was confirmed in SCC29B cells by Western blotting (Cropped blots are displayed; full-length blots are shown in Supplementary Figure S6).

Furthermore, IHC analysis demonstrated concurrent increase in protein expression of cIAP1 and cIAP2 across the transition from normal to OSCC via leukoplakia lesions (Fig. 2C). Interestingly, partially different subcellular localizations of cIAP1 and cIAP2 were observed in both normal and tumor cells, and were further confirmed by a pathologist. cIAP1 localized to the nucleus or the cytoplasm of tumor cells, whereas cIAP2 was seen in the membrane or in the cytoplasm of tumor cells, as depicted in Fig. 3A (a–d). In addition, based on correlation analysis, we found that the probabilities of nuclear cIAP1 and membranous cIAP2 protein expression were higher in normal and leukoplakia samples than in cancerous samples (Fig. 3B). Higher membranous cIAP2 protein expression was observed in well differentiated tumors, as well as in lymph node metastasis negative tumors (Supplementary Table S3). Moreover, cytoplasmic cIAP2 expression strongly correlated with node metastasis (Supplementary Table S3). Polytomous logistic regression with normal as the reference group showed a significant correlation between cIAP1 and cIAP2 cytoplasmic overexpression and the risk of developing OSCC (Supplementary Table S4). Overall, cytoplasmic overexpression of cIAP1 and cIAP2 was positively correlated with progression from normal tissue to OSCC.

### cIAP1 and cIAP2 expression in an OSCC cell line

**Figure 4:**
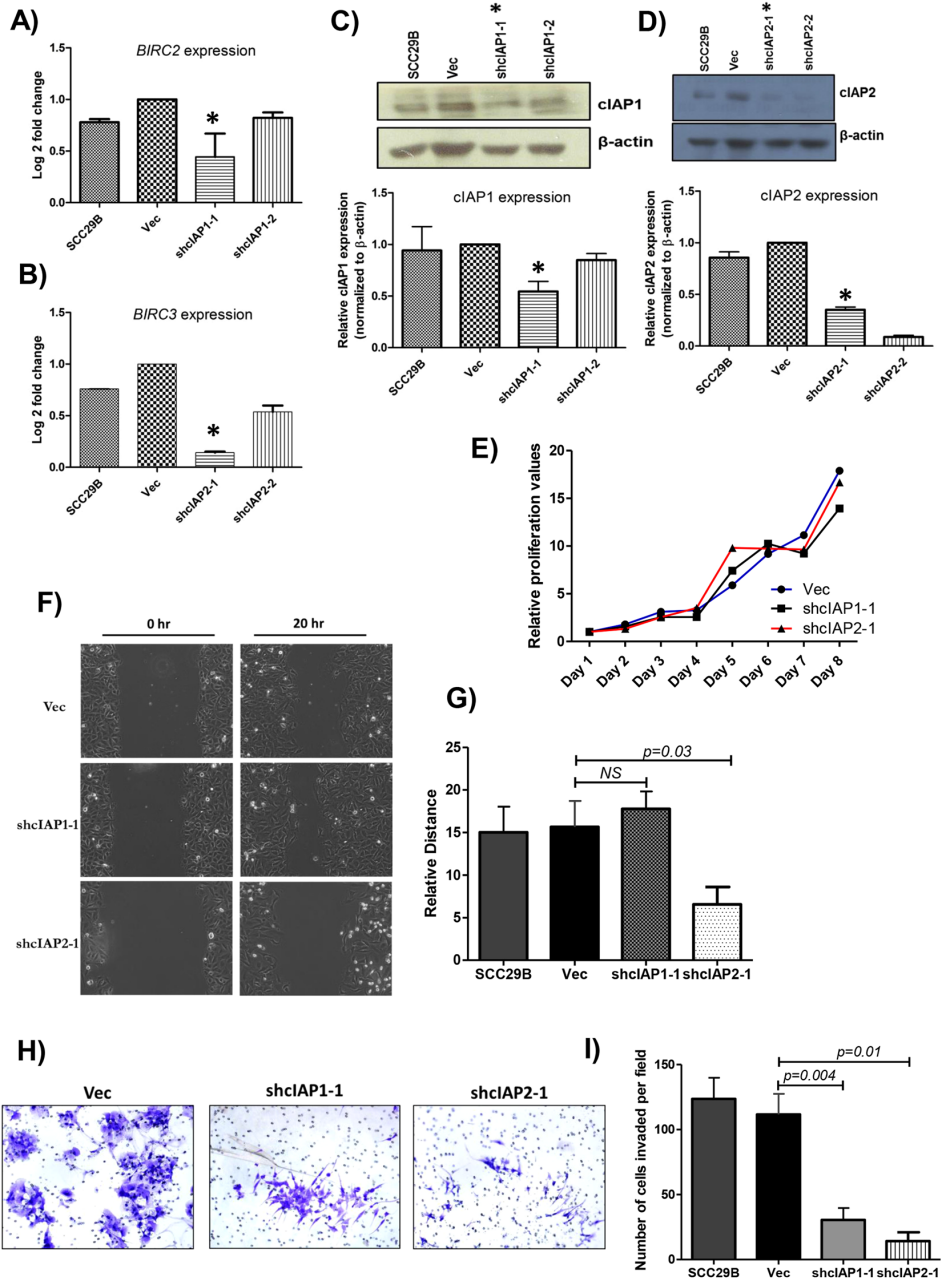
Effect of cIAP1 and cIAP2 knockdown on cell migration, invasion, and proliferation. Confirmation of cIAP1 and cIAP2 knockdown was done by (**A** and **B**) qRT-PCR and (**C** and **D**) Western Blotting. The histograms plot protein expressions after normalization with β-actin (Cropped blots are displayed; full-length blots are presented in Supplementary Figure S6). *Knockdown clones with decrease of either cIAP1 or cIAP2 at both mRNA and protein level were used for further experiments. (**E**) MTT-based cell proliferation assays were performed, and the relative difference in cell proliferation was determined over a period of 8 days in SCC29B-derived vector control (Vec), shcIAP1–1 and shcIAP2–1. No difference in cell proliferation was observed in knockdown cells as compared to Vec. (**F** and **G**) Scratch wound healing assays show a significant decrease in relative distance migrated in shcIAP2–1 cells, as compared to Vec. (**H** and **I**) Matrigel invasion assays showed reduced invasion in both shcIAP1–1 and shcIAP2–1 cells as compared to the Vec control (original magnification 100X). The mean ± standard deviations of three independent experiments are plotted. The *p* values were calculated using paired t-tests.

We examined both the expression and the localization of cIAP1 and cIAP2 in the buccal mucosa derived OSCC cell line UPCI:SCC029B (SCC29B), which has been reported to be aneuploid for chromosome 11^47^. We observed the upregulation of *BIRC2* and *BIRC3* in SCC29B when compared to normal gingivobuccal tissue (Fig. 3C). Comparable expression and similar localization of cIAP1 (nuclear and cytoplasmic) and cIAP2 (membranous and cytoplasmic) were observed in SCC29B, as seen in OSCC tumor samples (Fig. 3A (g,h), C and D). To understand the functional relevance of both these targets in SCC29B, we performed separate shRNA-mediated knockdowns of *BIRC2* and *BIRC3*. As SCC29B is a cell line which is difficult to transfect, we successfully generated three stable lines using only pooled populations (rather than single population cloning) for shcIAP1–1, shcIAP2–1, and pLKO.1-EGFP-f-puro vector (Vec). The observed transfection efficiency for the control Vec is shown in Supplementary Figure S4A. All the knockdowns were confirmed by qRT-PCR (Fig. 4A and B), Western blotting (Fig. 4C and D), and immunocytochemistry (ICC) (Supplementary Figure S4 B). Interestingly, we observed insignificant increases in the expression of cIAP1 and cIAP2 in Vec as compared to parental SCC29B cells (Fig. 4A–D). We speculate that the observed increases in expression could be because of the response of the cells to puromycin, which is used for selection. A study by Paek *et al*. also demonstrated the induction of IAP proteins in response to drugs such as cisplatin^48,49^.

### *BIRC3* promotes increased migration and invasion in OSCC

To examine the effect of cIAP1 and cIAP2 knockdown on cell migration, we performed scratch wound healing assays. We found that cell migration is decreased when cIAP2 is knocked down in SCC29B cells, but no effect was observed with cIAP1 knockdown (Fig. 4F and G). Furthermore, matrigel invasion assays showed a decrease in the invasive potential of cells upon either cIAP1 or cIAP2 knockdown (Fig. 4H and I), without any change in cell proliferation (Fig. 4E). These results suggest that cIAP2 is required for increased migration and invasion, while cIAP1 is required only for increased invasion in SCC29B.

### The effect of cIAP1 and cIAP2 on radioresistance

**Figure 5:**
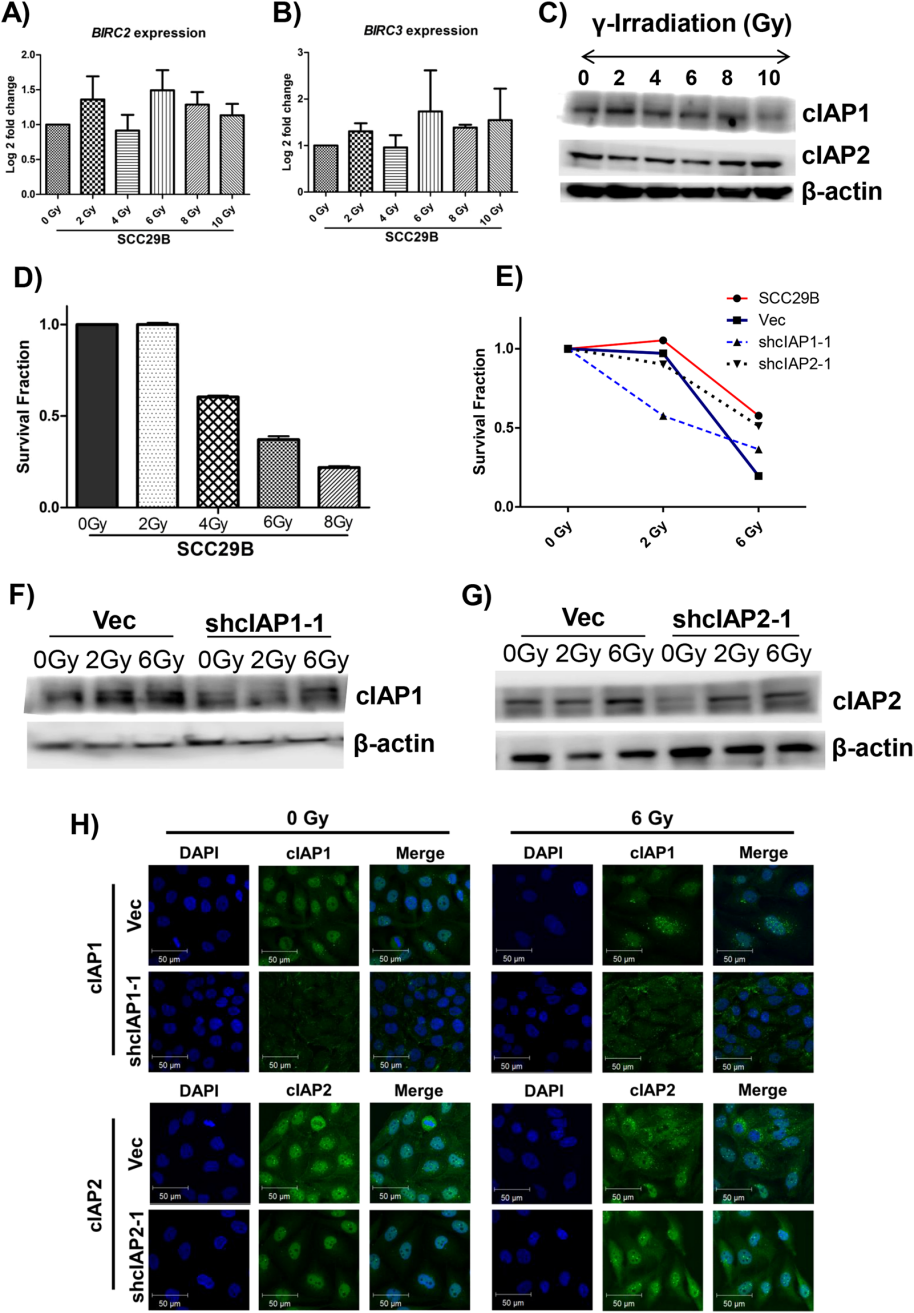
cIAP1 or cIAP2 knockdown had no effect on radioresistance. SCC29B cells were γ-irradiated with increasing dose, cells were harvested 24 hours post radiation, and expression of *BIRC2* and *BIRC3* were analyzed by (**A** and **B**) qRT-PCR and (**C**) Western blotting, as well as (**D**) clonogenic survival assay to calculate median lethal dose (LD50) of SCC29B. (**E**) The survival fraction of Vec and knockdowns was evaluated by clonogenic assay post γ-irradiation. (**F-G**) Protein expression of cIAP1 and cIAP2 was analyzed by Western blot and Immunocytochemistry (ICC) original magnification 630X at different radiation doses in Vec, shcIAP1–1 and shcIAP2-1 cells. Cropped blots are displayed (full-length blots and the histogram plots of the protein expressions after normalization with β-actin are presented in Supplementary Figure S6). The mean ± standard deviations of three independent experiments are plotted.

Clinical data showed that 27.9% of the analyzed OSCC patients had poor survival and 40.2% had recurrence, despite complete treatment, *i*.*e*., surgery followed by postoperative radiotherapy. Hence, we were interested in understanding the association of cIAP1 and cIAP2 with radioresistance in oral cancers. First, we quantified the expression of both cIAP1 and cIAP2 at the mRNA and protein levels with qRT-PCR and Western blotting and assessed the survival of SCC29B at different radiation doses (Fig. 5A–D, Supplementary Figure S5A). We observed an insignificant increase in the expression of *BIRC2* and *BIRC3* with increasing radiation dosage (Fig. 5B–D). Next, we assessed the effects of cIAP1 or cIAP2 knockdown on radiation response using colony formation assay (Supplementary Figure S5B) and observed no difference in survival of either cIAP1 or cIAP2 knockdown cells compared to vector (Fig. 5E). We speculated that this was a consequence of the increased cIAP1 and cIAP2 expression post irradiation, which was further confirmed by analyzing cIAP1 and cIAP2 expression levels in respective knockdowns at 6 Gy [Fig. 5F–H]. Specifically, we observed an increase in the expression of cIAP1 and cIAP2 in cells irradiated at 6 Gy, as compared to 0 Gy control, implying that the knockdown system was not sufficient to compensate for the radiation-induced overexpression.

## Discussion

Multiple aberrations on chromosome 11 have been strongly correlated with progression, metastasis, and radioresistance in HNSCC^8–10,50–53^. In the present study, we validated and confirmed the association of the 11q13.3 amplification with disease progression. Our data shows a strong correlation between the 11q22.2 amplification and lymph node metastasis and poor survival in HPV negative OSCC patients.

Using qRT-PCR and IHC, we demonstrated that the overexpression of *BIRC2* (cIAP1) and *BIRC3* (cIAP2), both located on 11q22.1-q22.2, was associated with lymph node metastasis in OSCC patients. Consistent with other reports, our study also highlights differences in localization of cIAP1 and cIAP2 in oral tumor tissues. cIAP1 is a tumor necrosis factor receptor (TRAF2)-related protein and localizes either to the nucleus or to the cytosol and shuttles between them^54–56^. When in the cytoplasm, it activates caspases in response to the apoptotic signal; while in the nucleus, it regulates cell cycle and cell proliferation^57^. cIAP2 has been reported to localize to the cytosol, peri-nucleus, and mitochondria^58,59^, and this study is the first to report the membranous localization of cIAP2 in OSCC. Cytoplasmic localization of the paralogous protein XIAP has been associated with increased cell survival and reduced apoptosis^60^, which is consistent with the notion that the increased cytoplasmic cIAP1 and cIAP2 expression that we observed in OSCC may promote tumor survival. The mechanisms controlling the distribution of cIAPs into various subcellular locations and their functional relevance in particular locations require further investigation^55^.

The expression of cIAP1/cIAP2 has been correlated with invasion in esophageal, cervical, and gall bladder cancers^61–63^. The current study demonstrates the role of cIAP1 and cIAP2 in invasion or migration in a buccal mucosa-derived OSCC cell line, further confirming the role of these proteins in lymph node metastasis in oral cancers. However, additional studies are required to validate the present cIAP1 and cIAP2 findings in additional OSCC and HNSCC cell lines before implicating these biomarkers in outcome prediction. The overexpression of cIAP1 can lead to genomic alterations due to defects in cell division and is a key regulator of cell proliferation and apoptosis^55^. Similarly, cIAP2 is also involved in regulating apoptotic signals, and the knockout of cIAP1 has been demonstrated to increase cIAP2 expression to compensate for cIAP1 loss, indicating that each of cIAP1 and cIAP2 may be redundant if the other one is functional^11^. Further studies might assess the synergistic effects of cIAP1 and cIAP2 on invasion and metastasis.

Previous reports support the fact that the overexpression of cIAP1/2 proteins may be associated with an unfavorable prognosis after radiotherapy or chemotherapy^14,40,54,64^. Here, we observed an increase in cIAP1 and cIAP2 expression in radiation-treated cells, as compared to their respective controls. This result is consistent with the study by Wang *et al*. reporting an increased expression of *BIRC3* in response to radiotherapy and TMZ treatment in glioblastoma^12^. The knockdown system may not be sufficient to demonstrate the effect of cIAP1 and cIAP2 on radiation response. Further studies are needed to better understand their role on radiation resistance in SCC29B, as well as in other OSCC cell lines, using knockout systems. Since therapy resistance is an important factor in determining patient outcome, the use of SMAC mimetics that inhibit IAPs could improve the efficacy of standard therapy and may emerge as a potential therapy for various cancers including OSCC^14,25,38^.

The current study highlights the importance of 11q22 locus amplification as a clinically relevant marker for predicting lymph node metastasis and poor clinical outcome, *i*.*e*., increased recurrence and reduced survival. We also report a correlation between the 11q22.1–22.2 amplification and poor radiation response, which could be further explored to modulate treatment regime for oral cancer patients. For the first time, we demonstrate the localization of cIAP2 to the cell membrane. The cytoplasmic localization of cIAP1 and cIAP2 was frequently observed in tumors, while nuclear cIAP1 and membranous cIAP2 were predominantly observed in normal and well differentiated tumors. Further investigations are needed to understand the molecular mechanisms underlying differential localization and therapeutic potential of cIAP1 and cIAP2 in oral cancers.

## Methods

### Tissue specimen collection

The study was approved by the Institutional Local Ethics Committee of Tata Memorial Hospital (TMH) and Nair Hospital Dental College, Mumbai, India. Paraffin blocks or frozen tissue samples of neo-primary oral tumor tissues and pre-invasive lesions (leukoplakia) were recruited from ICMR National Tumor Tissue Repository, Tata Memorial Hospital. Non-inflamed gingivobuccal mucosa tissues from clinically healthy individuals with no previous personal history of cancer were obtained from Nair Hospital. The methods were carried out in accordance with the approved guidelines and regulations. Written informed consent was obtained from all the study participants.

### Cell line and plasmid constructs

The oral squamous cell carcinoma cell line UPCI:SCC029B (SCC29B), derived from human buccal mucosa cancer, was procured from Dr. Susanne M. Gollin, The University of Pittsburgh, USA^65^. SCC29B cells were cultured in M10 media, as recommended by the supplier. The plasmids containing shRNAs against cIAP1 and cIAP2 were a gift from William Hahn (Addgene plasmid)^66^; the specific plasmids we used are denoted here as shcIAP1–1 and shcIAP2–1. The pLKO.1-EGFP-f-puro vector without any shRNA was used as vector control (denoted here as Vec). The sequences for the cIAP1 and cIAP2 shRNAs are provided in Supplementary Table S5. The plasmids were purified on a cesium gradient and the presence of the shRNA insert was confirmed by sequencing.

### Real-Time PCR (qRT-PCR) Analysis

A total of 10 µl RNA (1.5 µg) were converted to cDNA using the High Capacity cDNA Reverse Transcription Kit (Applied Biosystems, USA), as described by the manufacturer. Following conversion, 6 ng of cDNA were used for TaqMan qRT-PCR analysis using fluorescent TaqMan probes obtained from Applied Biosystems for *BIRC2* (Hs01112284_m1), *BIRC3* (Hs00985031_g1), and *RNA18S5* (Hs99999901_s1) as a control. All the experiments were performed in duplicate and the results were analyzed using QuantStudio 12 K Flex software v1.2.2 (Applied Biosystems, USA), as described previously^6^.

### Interphase/Nuclear *in-situ* Hybridization (nuc ish)

Nuc ish was performed using 1) BAC probes (BACPAC resource center, Children’s Hospital Oakland Research Institute, USA) for the 11q13.3 locus (RP11–300I6) and the Chr11 centromere (CEP) (RP11–135H8) and 2) SureFISH probes (Agilent Technologies, USA) for the 11q22.1–11q22.2 locus (G101223G-8) and the Chr11 CEP (G101083R-8). The specificities of all the BAC-derived probes and sureFISH probes were confirmed on metaphase target slides (Vysis, CA, US) before hybridizations. Visualization and enumeration of nuc ish signals was done as described previously^6^. A *weak amplification* was defined as having the gene probe counts between 3 and 8. *Strong amplification* was defined as having either gene probe counts greater than 8 or innumerable clusters for the gene locus. For statistical analyses, two types of comparisons were done: 1) no change (diploid nuclei) vs. change (includes cases with either weak amplification or with strong amplification); 2) no change vs. strong amplification (excluding cases with weak amplification).

### Immunohistochemistry (IHC)

Immunohistochemical staining, grading, and H-Score analysis were performed as described previously^6^. In this study, IHC was performed for cIAP1 and cIAP2 using Vectastain Universal Elite ABC Kit (Vector Labs, USA) or Dako EnVision™ FLEX Mini Kit, High pH kit (DAKO, Agilent Technologies, Denmark). Details on antibodies and heat-based antigen retrieval are provided in Supplementary Table S6.

### Immunofluorescence (IF) and Immunocytochemistry (ICC)

Immunofluorescence analysis was performed on tissue samples and SCC29B cells. For tissue preprocessing and antigen retrieval, the steps were performed as described for IHC, followed by tissue fixing in cold methanol for 10 minutes, followed by blocking. Tissues were next incubated for 16 hours at 4 °C with the respective antibody at a dilution provided in Supplementary Table S6. On the following day, the slides were incubated with an Alexa Fluor 488 anti-rabbit secondary antibody (Life Technologies, USA) at 1:200 dilutions, in a humid chamber for 1 hour at room temperature.

For ICC, SCC29B cells were grown on a coverslip; after the cells reached 80% confluency, the cells were fixed in 4% paraformaldehyde and then permeabilized using 0.3% Triton-X100 for 20 minutes at room temperature. Following blocking, the cells were treated with primary antibodies inside a humidified chamber for 16 hours (overnight) at 4 °C. On the following day, the cells were treated with the Alexa 488 or Alexa 568 conjugated secondary antibody anti rabbit IgG (Life Technologies, USA) at a dilution of 1:100 and incubated for 1 hour at room temperature in a humidifying container. Both the tissues and the cells were treated with nuclear counterstain 4′,6-Diamidine-2′-phenylindole dihydrochloride (DAPI) and then mounted using the Vectashield mounting agent (Vector Laboratories, USA). Confocal images were obtained by using an LSM 780 Carl Zeiss Confocal system with an Argon 488 nm, 568 nm, and 405 nm lasers (Carl Zeiss, Germany), and image analysis was performed using the LSM image browser.

### Transfection of SCC29B cells

Transfection was done in a 12-well plate with 1 μg of plasmid DNA, using Lipofectamine™ 3000 Reagent (Invitrogen, USA), and carried out as per manufacturer’s protocol, at 70–90% cell confluency. Stable lines including each of the two shRNAs to knock down cIAP1 and cIAP2 were generated by using a pool of all cell populations that survived puromycin selection (0.75 μg/ml).

### Western blotting

For Western blots, 50 μg of the whole cell lysate was resolved on 10% SDS-PAGE gel and transferred to PVDF membranes (Amersham Hybondtm-P, GE Healthcare, USA), followed by Western blotting with antibodies for cIAP1 and cIAP2 (antibody details are provided in Supplementary Table S6). The blots were developed using Amersham ECL Prime Western Blotting Detection Reagent (GE Healthcare, USA), according to the manufacturer’s instructions and quantified with Image J software.

### Scratch wound healing assays

Cells were grown to 90% confluency in a 6-well plate, followed by treatment with 10 μg/ml of mitomycin C (Sigma-Aldrich, USA) for 3 hours. Subsequently, the cells were washed and a linear scratch wound was made in each well. The cells were maintained at 37 °C and 5% CO_2_ on an Axiovert 200 M Inverted microscope (Carl Zeiss, Germany) with a cell incubator stage. Cells were observed by time lapse microscopy and images were taken every 10 minutes for 20 hours, using a AxioCamMRm Camera (Carl Zeiss, Germany) with a 10X phase I objective.

### Matrigel cell invasion assay

Matrigel invasion assays were performed in a 24-well plate using 2 × 10^5^ cells re-suspended in 200 μl of serum-free media. These cells were added to the upper chambers and 600 μl of conditioned media (1:1 ratio of conditioned and fresh serum containing media) were added in the lower chamber. The inner side of the insert with 0.8 mm membrane (BD Falcon, USA) was pre-coated with 15 μl of Matrigel (Corning, USA). After 24 hours, the inserts with cells on the outer side of the membrane were fixed with 4% para-formaldehyde, stained with 1% crystal violet (Sigma-Aldrich, USA), and mounted on slides using D.P.X mounting reagent (Merck, USA). Images were taken using an Olympus SZ61 stereo microscope with a 10X objective.

### Cell proliferation assay

An MTT-based assay was used to measure cell proliferation in 96-well plates on 5000 cells for eight days. At 24 hour intervals, cells were treated with 20 µl MTT (3-(4, 5-dimethylthiazolyl-2)-2, 5-diphenyltetrazolium bromide) (Sigma-Aldrich, USA) for 4 hours at 37 °C in a CO_2_ incubator. The reaction was stopped using 100 µl stop solution (10% SDS in 0.01 N HCL), followed by overnight (16 hours) incubation at 37 °C. This assay measures cell metabolic activity by comparing optical densities between the end and the start of the experiment. Absorbance was read at 530 nm and 690 nm using a microplate reader, SPECTROstar^Nano^ (BMG Labtech, Germany).

### Clonogenic cell survival assay

Clonogenic survival was determined at radiation doses of 0, 2, 4, 6, and 8 Gy for the original SCC29B and 0, 2 and 6 Gy for the three derived clones with a control vector (Vec) plasmid, a cIAP1 knockdown plasmid, and a cIAP2 knockdown plasmid. Cells from the exponential growth phase were γ-irradiated using ^60^Co-γ Linear Accelerator (Bhabhatron-2, ACTREC, Tata Memorial Centre), and then seeded in triplicate in 60 mm plates. Colonies were allowed to grow for 10 days before being stained with 0.05% crystal violet. The survival fraction at a given dose was calculated as the number of colonies formed divided by the product of the number of cells plated and plating efficiency.

### Statistical analyses

The statistical analyses were performed using IBM SPSS version 21. Chi-square tests or Spearman correlation tests were used to determine the correlation between protein expression levels or locus amplification and disease progression, as well as clinicopathological characteristics such as lymph node metastasis, tumor stage, and grade. Multinomial logistic regression was used to evaluate the relationship between protein expression scores and the risk of leukoplakia and OSCC development, with normal tissue as a reference; odds ratios (OR) were computed by adjusting for age and gender^67,68^. Disease-specific survival (DSS) and recurrence free survival (RFS) were examined visually with Kaplan-Meier curves and analyzed by log rank tests. Binomial logistic regression was performed to analyze the dependence of 11q13.3 and 11q22.1-q22.2 amplifications on lymph node metastasis using either 1) cases with both loci altered, 2) cases with only 11q13.3 altered and no change at 11q22.1-q22.2, or 3) cases with only 11q22.1-q22.2 altered and no change at 11q13.3. Multivariate Cox regression was performed to identify the degree of association between various parameters such as lymph node metastasis, tumor stage, grade, and clinical outcome. All *in vitro* assays were performed in triplicate. For cell line experiments, paired t-tests were used to analyze the relationships between vector control and cIPA1 and cIAP2 knockdown clones. All p-values < 0.05 were considered statistically significant.

## Electronic supplementary material

Supplementary Information
