# Supplementary material for: Recurring Amplification at 11q22.1-q22.2 Locus Plays an Important Role in Lymph Node Metastasis and Radioresistance in OSCC
Source: Sci Rep. 2017 Nov 22;7:16051. doi: 10.1038/s41598-017-16247-y (PMC5700126; doi:10.1038/s41598-017-16247-y)
Supplement: Supplementary file 1 — Supplementary Information [file 41598_2017_16247_MOESM1_ESM.pdf]

Supplementary Information

Recurring Amplification at 11q22.1-q22.2 Locus Plays an Important Role in Lymph Node Metastasis and Radioresistance in OSCC

Priyanka G. Bhosale[1,2], Manishkumar Pandey[1,2], Simona Cristea[3,4,5,6], Mickey Shah[1], Asawari Patil[7], Niko Beerenwinkel[3,4], Alejandro A. Schäffer[8], Manoj B. Mahimkar[1,2,*]

1: Cancer Research Institute (CRI), Advanced Centre for Treatment, Research and Education in Cancer (ACTREC), Tata Memorial Centre (TMC), Navi Mumbai-410210, India

2: Homi Bhabha National Institute, Training School Complex, Anushakti Nagar, Mumbai-400085, India

3: Department of Biosystems Science and Engineering, ETH Zurich, 4058 Basel, Switzerland

4: SIB Swiss Institute of Bioinformatics, 4058 Basel, Switzerland

5: Department of Biostatistics and Computational Biology, Dana-Farber Cancer Institute, Boston MA 02115

6: Department of Biostatistics, Harvard School of Public Health, Boston MA 02115

7: Department of Pathology, Tata Memorial Hospital, Tata Memorial Centre (TMC), Parel, Mumbai-400012, India

8: Computational Biology Branch, National Center for Biotechnology Information, National Institute of Health (NIH), Department of Health and Human Services (DHHS), Bethesda, Maryland, 20894, USA

***Correspondence to: mmahimkar@actrec.gov.in**

Supplementary Table S1: Frequency of 11q13.3 and 11q22.1-q22.2 alterations in OSCC.

**Alteration**	**11q13.3 *n* (%)**	**11q22.1-q22.2 *n* (%)**
**Change**	**72 (53)**	**60 (37.5)**
Weak amplification	*49 (69)*	*41 (68)*
Strong amplification	*23 (31)*	*19 (32)*
**No change (diploid nuclei)**	**71 (47)**	**100 (62.5)**

n represents the number of samples

Supplementary Table S2: Association of 11q13.3 & 11q22.1-q22.2 alterations with clinicopathological parameters.

	11q13.3 alteration				
**Clinicopathological parameter**	**11q13 no change**	**11q13 change‡**	***p** Value**	**11q13 strong amplification**	***p** Value**
**Pathological stage**					
Stage 1 and 2 (Early stage OSCC)	18	6	**0.006**	0	**0.003**
Stage 3 and 4 (Advanced stage OSCC)	53	66		23	
**Pathological grade**					
Well	6	3	0.37	1	0.27
Moderate	49	47		13	
Poor	16	22		9	
**Lymph node metastasis**					
Negative (N0)	45	27	**0.002**	7	**0.006**
Positive (N+)	26	45		16	

	11q22.1-q22.2 alteration				
**Clinicopathological parameter**	**11q22 no change**	**11q22 change‡**	***p** value**	**11q22 strong amplification**	***p** value**
**Pathological stage**					
Stage 1 and 2 (Early stage OSCC)	23	8	0.15	2	0.35
Stage 3 and 4 (Advanced stage OSCC)	77	52		17	

**Pathological grade**					
Well	9	2	0.10	0	**0.018**
Moderate	72	39		10	
Poor	19	19		9	
**Lymph node metastasis**					
Negative (N0)	66	18	**<0.0001**	5	**0.002**
Positive (N+)	34	42		14	

‡: Change: Includes both weak and strong amplification; *Two tailed Fisher's exact test; 11q13:11q13.3; 11q22: 11q22.1-q22.2

Supplementary Table S3: Correlation of cIAP1 and cIAP2 expression with clinicopathological parameters.

	Clinicopathological parameters	
	**Nodal metastasis**	**Grade**
**cIAP1 cytoplasmic expression**	p=0.33	p=0.20
**cIAP1 nuclear expression**	p=0.34	p=0.46
**cIAP2 cytoplasmic expression**	***p*=0.027**	p=0.30
**cIAP2 membranous expression**	****p*=0.049**	****p*=0.005**

*: positive correlation; **: negative correlation; *p* is the p-value

Supplementary Table S4: The Effect of cIAP1 and cIAP2 overexpression on OSCC development.

	**Normal (reference)**	**Leukoplakia**		**OSCC**	
		***p** value**	**OR (95% CI)**	***p** value**	**OR (95% CI)**
**cIAP1 cytoplasmic expression**	1	0.55	1.013 (0.971-1.05)	**0.003**	1.07 (1.02-1.13)
**cIAP1 nuclear expression**	1	0.50	1.008 (0.985-1.03)	**0.006**	0.94 (0.89-0.98)
**cIAP2 cytoplasmic expression**	1	0.52	1.007 (0.985-1.03)	**0.04**	1.02 (1.00-1.05)
**cIAP2 membranous expression**	1	0.54	1.005 (0.989-1.02)	0.78	1.00 (0.98-1.03)

OR: Odds Ratio, * Multinomial logistic regression; *p*: p-value

Supplementary Table S5: cIAP1 and cIAP2 shRNA sequence.

Gene	Plasmid from Addgene	shRNA sequence
BIRC2/cIAP1	pLKO-shcIAP1-A (Addgene plasmid # 44129)	GCCGAATTGTCTTTGGTGCTT
	pLKO-shcIAP1-B (Addgene plasmid # 44131)	CAGTTCGTACATTTCTTTCAT
BIRC3/cIAP2	pLKO-shcIAP2-A (Addgene plasmid # 44130)	GCTGCGGCCAACATCTTCAAA
	pLKO-shcIAP2-B (Addgene plasmid # 44132)	GCACTACAAACACAATATTCA

Supplementary Table S6: Details of antibodies used in the study.

**Target protein & antibody detail (Catalog)**	**IHC Method (kit)**	**Retrieval method**	**Retrieval buffer**	**Retrieval time**	**IHC antibody dilution and diluent**	**IF/ICC antibody dilution**	**Immuno-blot antibody dilution**
**<u>cIAP1;</u>** abcam (catalog no: ab2399)	Dako EnVision™ FLEX Mini Kit, High pH	Waterbath at 100°C followed by microwave	EnVision™ FLEX Target Retrieval Solution, High pH (Cat#DM828)	20 min (15 min waterbath and 5 min microwave)	1:200, 1x PBS	Not used for ICC or IF	Not used for WB
**<u>cIAP1;</u>** Novus Biologicals (catalog no: NB100-56128)	Not applicable	Not applicable	Not applicable	Not applicable	Not used for IHC	(1:50)	(1:500)
**<u>cIAP2;</u>** Novus Biologicals (catalog no: NBP1-27972)	Vectastain Universal elite ABC kit	Microwave	EDTA buffer pH 8	12 min (6 min twice)	1:1000, 1% serum+ 1x PBST	(1:100)	(1:1000)

Supplementary Figures

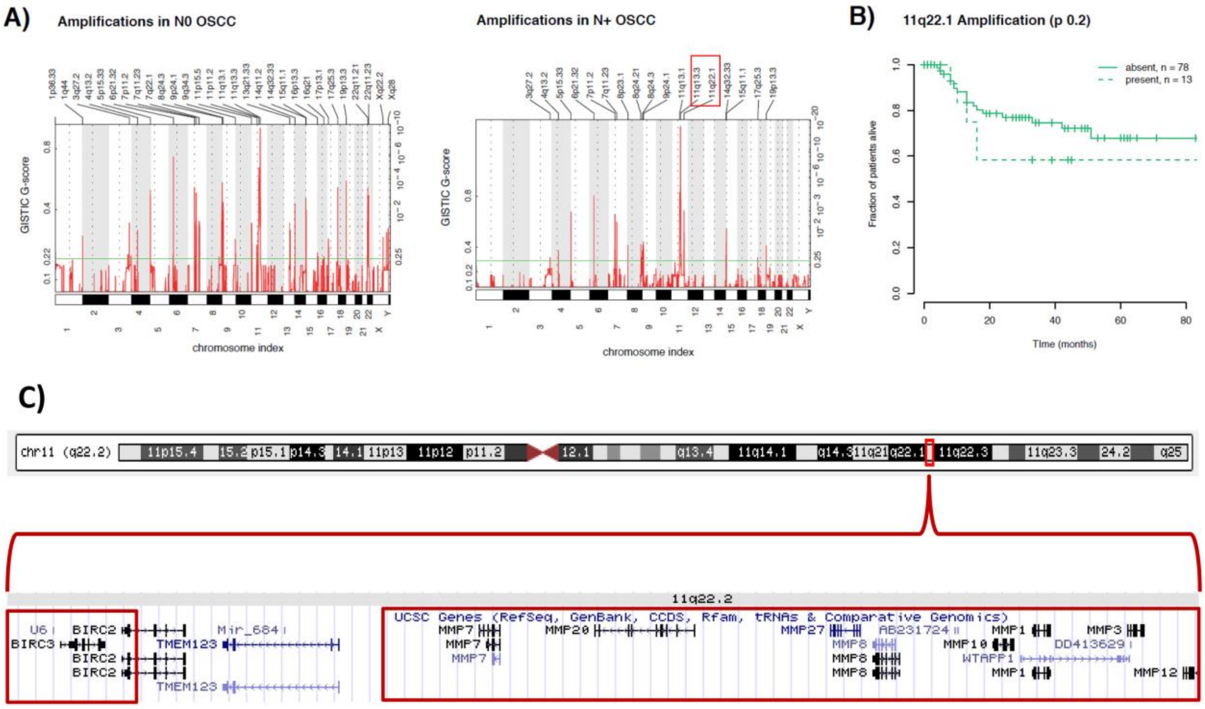

Supplementary Figure S1: Amplification of 11q22.1 has been observed in lymph node metastasis and is significantly associated with patient survival [6]. A) Amplifications inferred by GISTIC 2.0 in node negative (N0) and node positive (N+) OSCC samples. Each aberration is assigned a G-score (Y axis) by GISTIC. **B)** Kaplan-Meier plots of disease specific patient survival for 11q22.1 amplification. *p* represents the p-value. **C**) Genetic map of 11q22.1-q22.2 loci with target genes.

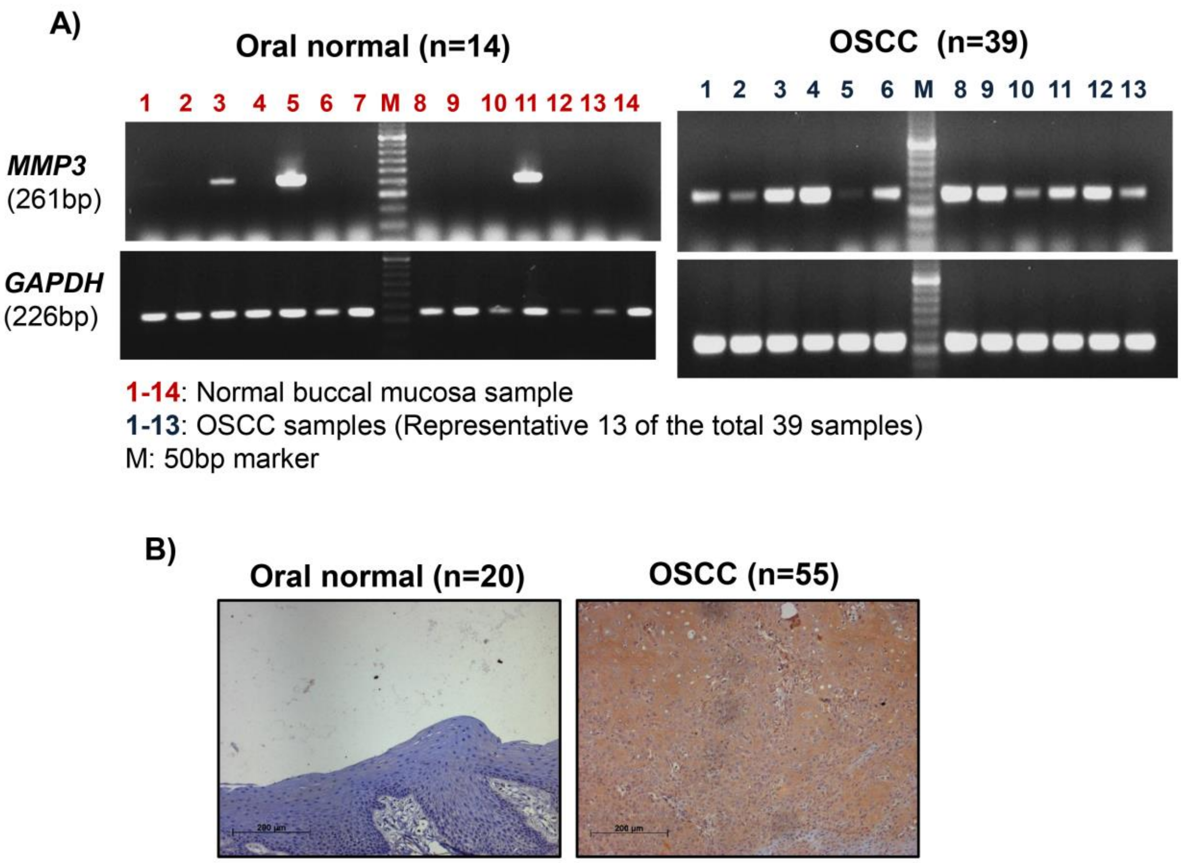

Supplementary Figure S2: Expression of MMP3 in oral normal and cancerous patient samples. A) *MMP3* gene expression analysis using semi quantitative RT-PCR using *GAPDH* as reference in normal and OSCC. The following primers were used for *MMP3* (Forward: AGCAAGGACCTCGTTTTCATT; Reverse: GTCAATCCCTGGAAAGTCTTCA) and *GAPDH* (Forward: GAAGGTGAAGGTCGGAGTC; Reverse: GAAGATGGTGATGGGATTTC). **B)** The representative IHC staining depicting MMP3 protein expression in normal and OSCC at 100X original magnification. n represents the number of patient samples used for each experiment.

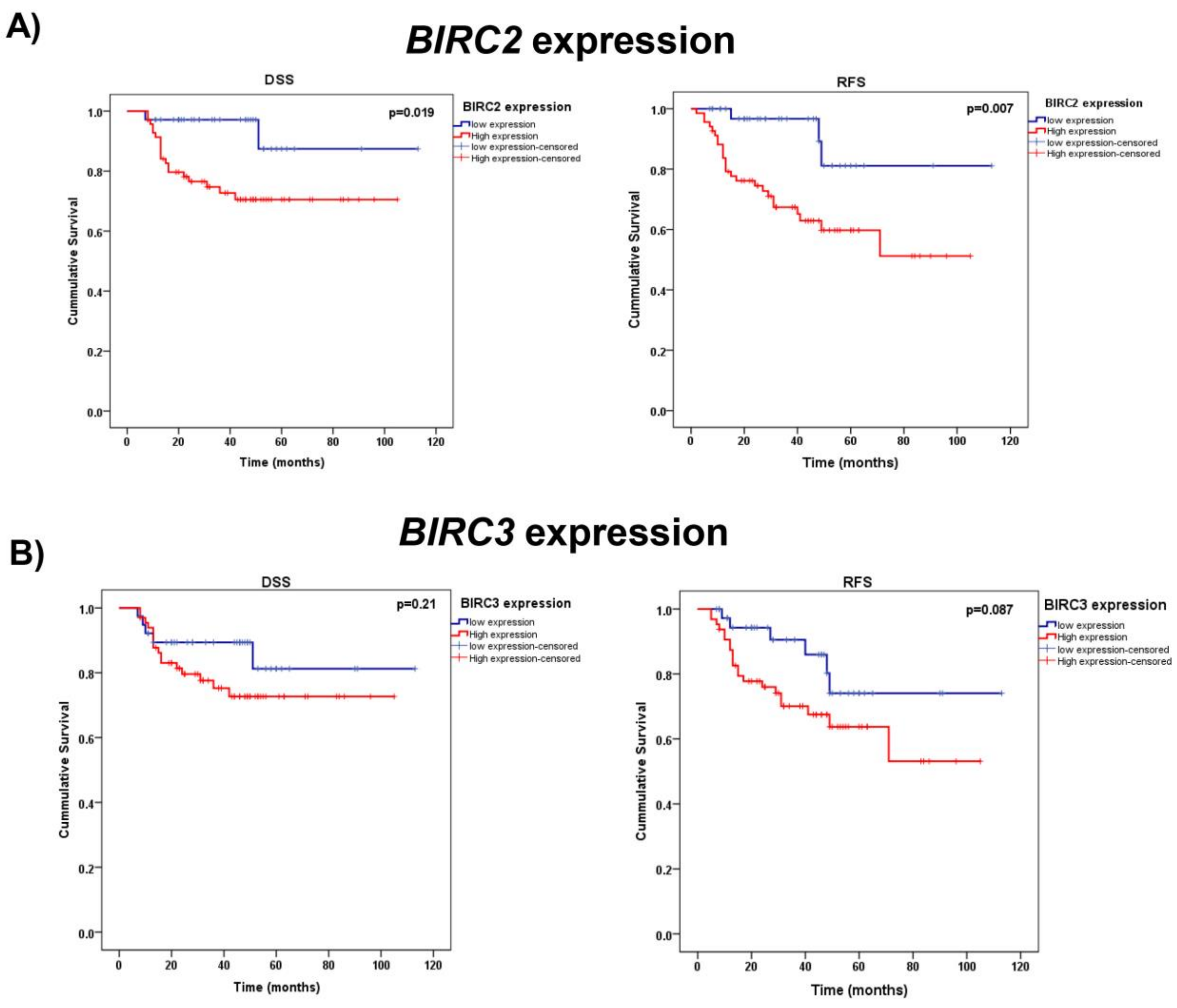

Supplementary Figure S3: Kaplan-Meier plots of patient survival for *BIRC2* and *BIRC3* upregulation. A) disease-specific survival (DSS) and recurrence-free survival (RFS) of patient groups with *BIRC2* gene upregulation. **B)** DSS and RFS of patient groups with *BIRC3* gene upregulation. DSS/RFS in months (x-axis) is plotted against the fraction of samples alive/ without recurrence (y-axis).

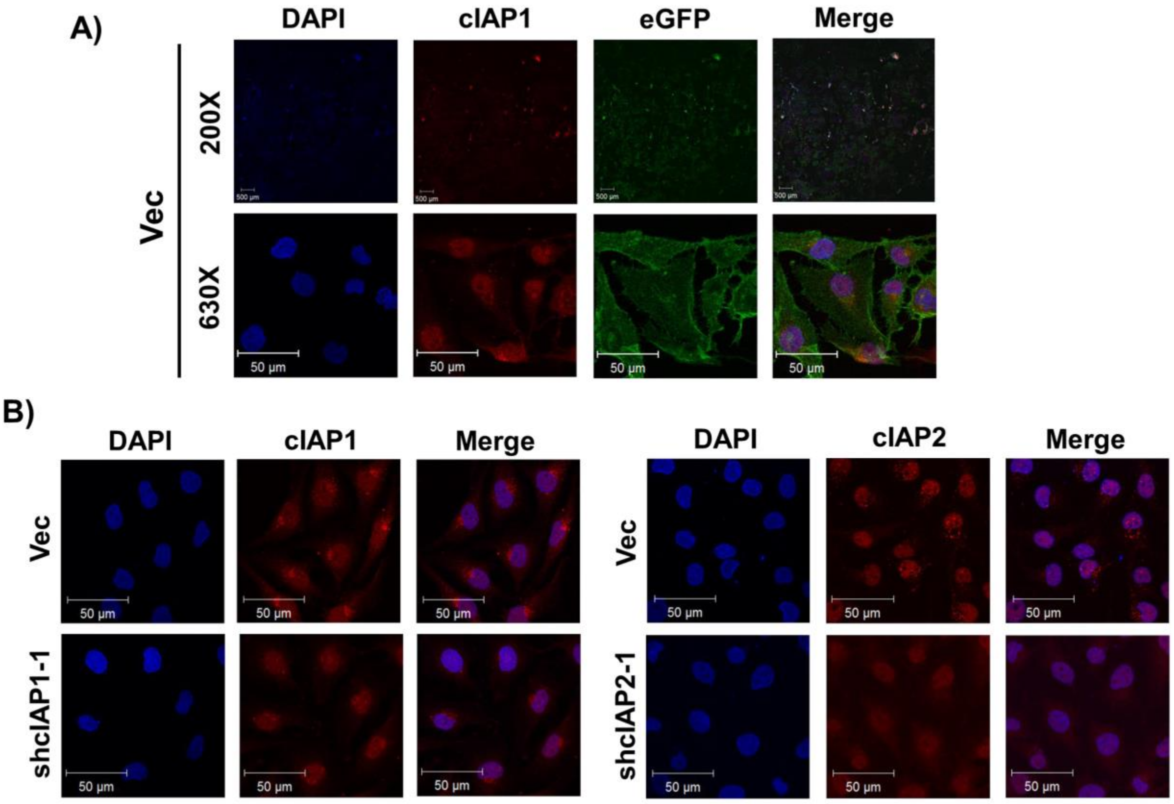

Supplementary Figure S4: Transfection of SCC29B cells. A) Transfection efficiency of SCC29B cells as verified by ICC in Vec. Cells were stained with the nuclear stain DAPI (blue, first panel), as well as cIAP1 primary antibodies and Alexafluor 568 secondary antibodies (red, second panel). eGFP staining confirms the presence of pLKO.1-EGFP-f-puro vector (green, third panel), and the fourth panel is a merge of first three panels. **B)** A decrease in cytoplasmic cIAP1 and cIAP2 was confirmed by ICC in respective knockdown lines. Cells were stained with the nuclear stain DAPI (blue, first panel), as well as with cIAP1/cIAP2 primary antibodies and Alexafluor 568 secondary antibodies (red, second panel). The last panel is the merged image of the first two panels (original magnification 630X).

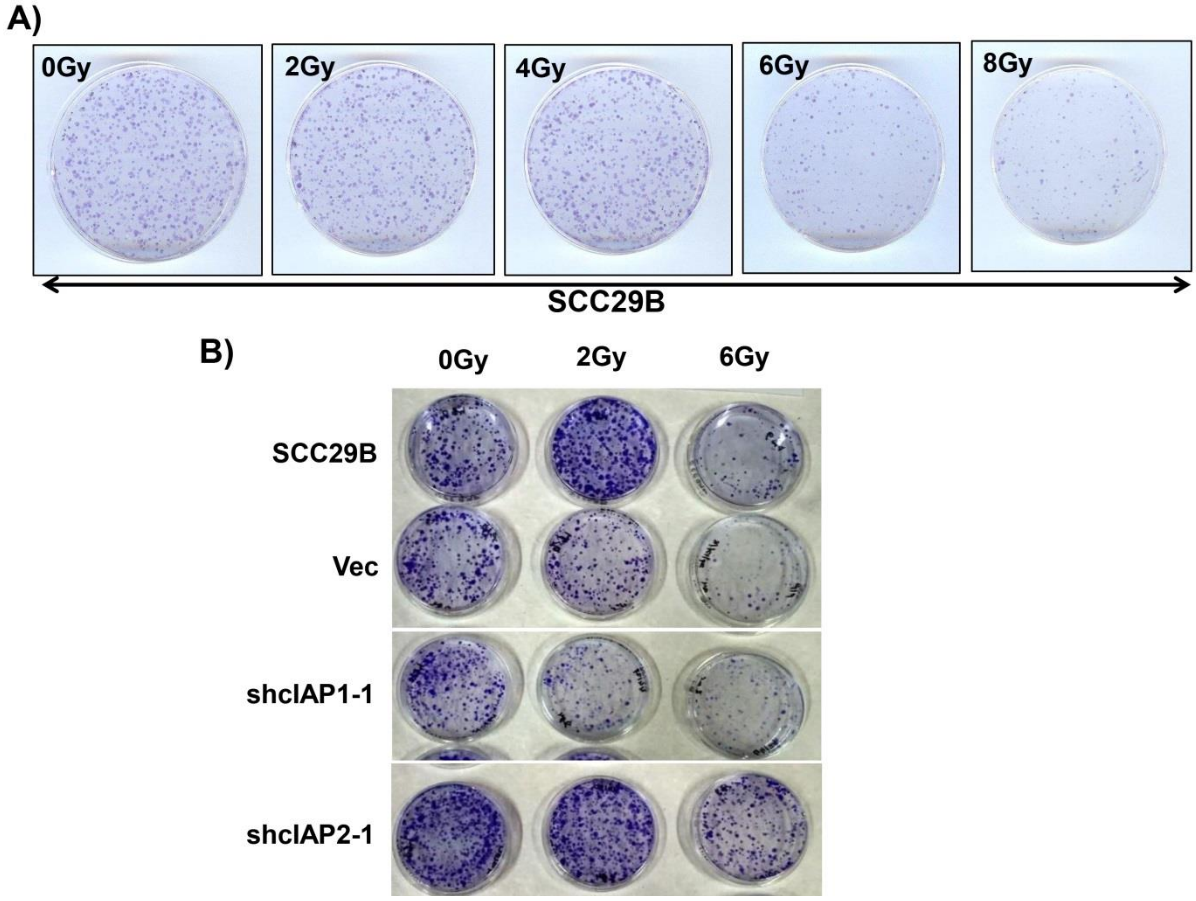

Supplementary Figure S5: Representative images of clonogenic cell survival assay. A) Colonies generated after γ-irradiating SCC29B cells with increasing radiation dose. **B)** Colonies generated after γ-irradiating SCC29B, Vec, shcIAP1-1 and shcIAP2-1 cells with 0, 2, and 6Gy radiation dose.

Supplementary Figure S6: Full length blots for Figure 3D, Figure 4C and 4D, Figure 5C, 5F, 5G.

S6-1: Representative Ponceau stained blot along with molecular weight marker depicting the positions at which blots where cut before probing with respective primary antibody.

S6-2: Figure 3D along with respective full length blot, developed on ProMax Xray Film Processor using Xray films exposed between 15 sec to 2 min. (Note: cIAP1; abcam antibody was used only for this blot).

S6-3: Figure 4C along with respective full length blot, developed on ProMax Xray Film Processor using Xray films exposed between 15 sec to 2 min.

S6-4: Figure 4D along with respective full length blot, developed on ProMax Xray Film Processor using Xray films exposed between 15 sec to 2 min.

S6-5: Figure 5C along with respective full length blot, developed on C-digit blot scanner (LI-COR) with a pre-set auto exposure time of 10 min. The histograms plot protein expressions after normalization with β-actin.

S6-6: Figure 5F and 5G along with respective full length blot, developed on C-digit blot scanner (LI-COR) with a pre-set auto exposure time of 10 min. The histograms plot protein expressions after normalization with β-actin.

S6-1

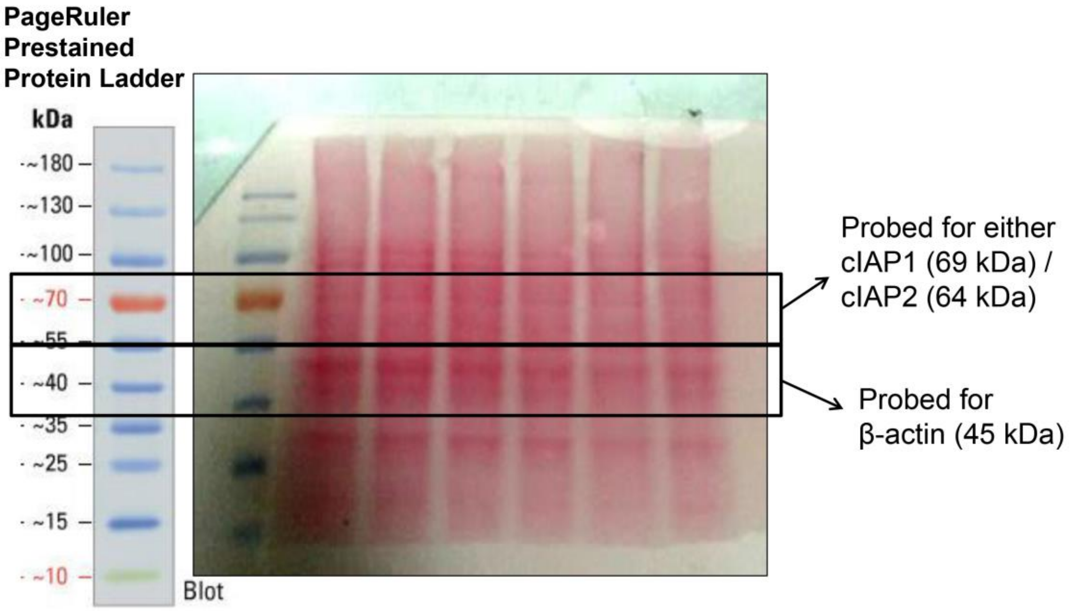
PageRuler
Prestained
Protein Ladder
kDa
~180
~130
~100
~70
~55
~40
~35
~25
~15
~10
Blot
Probed for either
cIAP1 (69 kDa) /
cIAP2 (64 kDa)
Probed for
β-actin (45 kDa)

S6-2

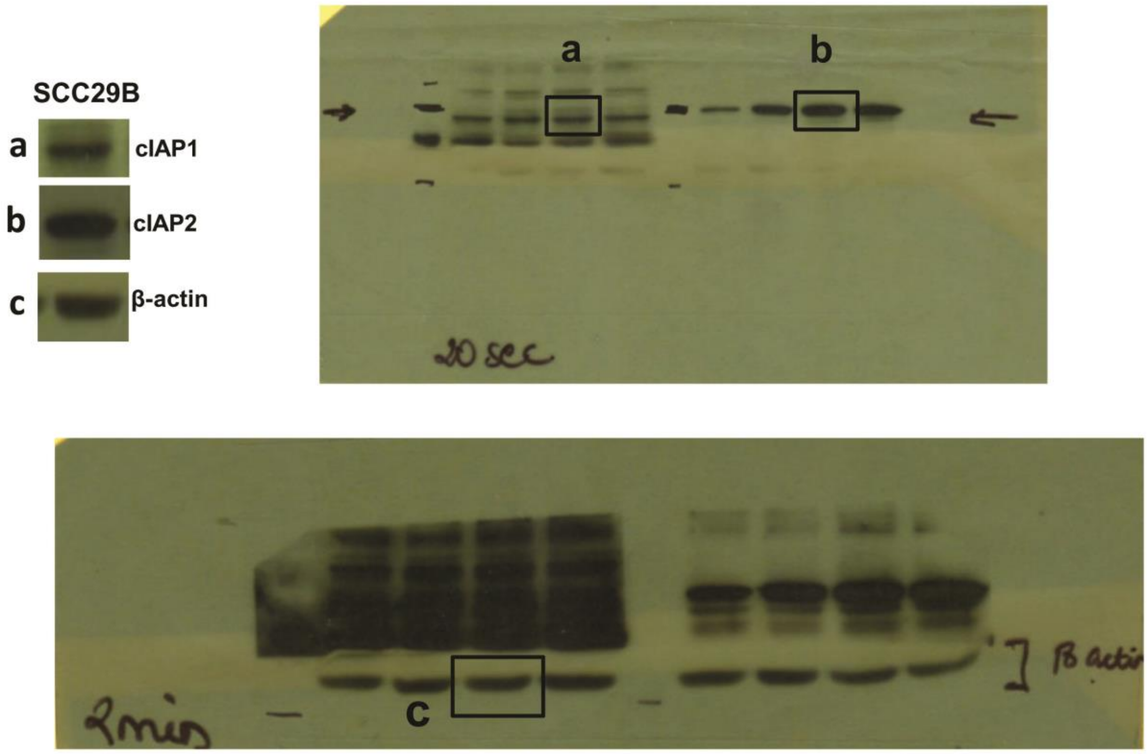
SCC29B
a
cIAP1
b
cIAP2
c
β-actin
a
b
20 sec
c
2min

S6-3

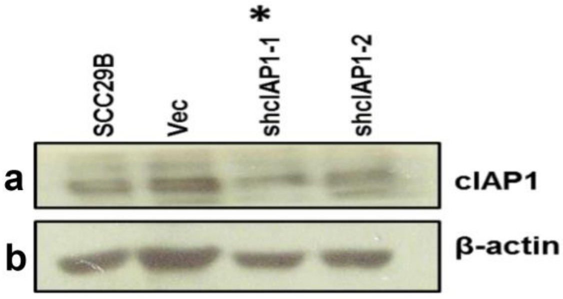

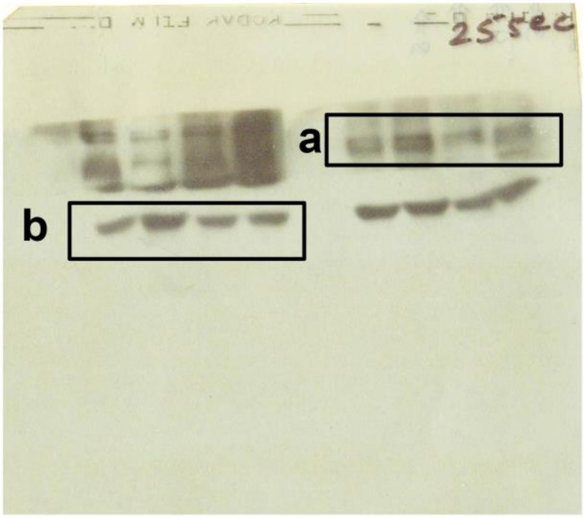

S6-4

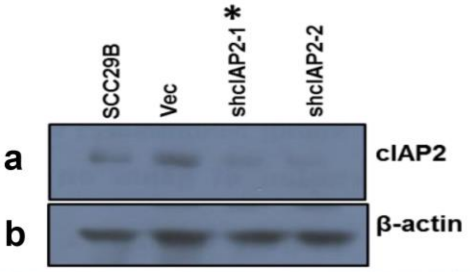
SCC29B
Vec
shcIAP2-1 *
shcIAP2-2
a
b
cIAP2
β-actin

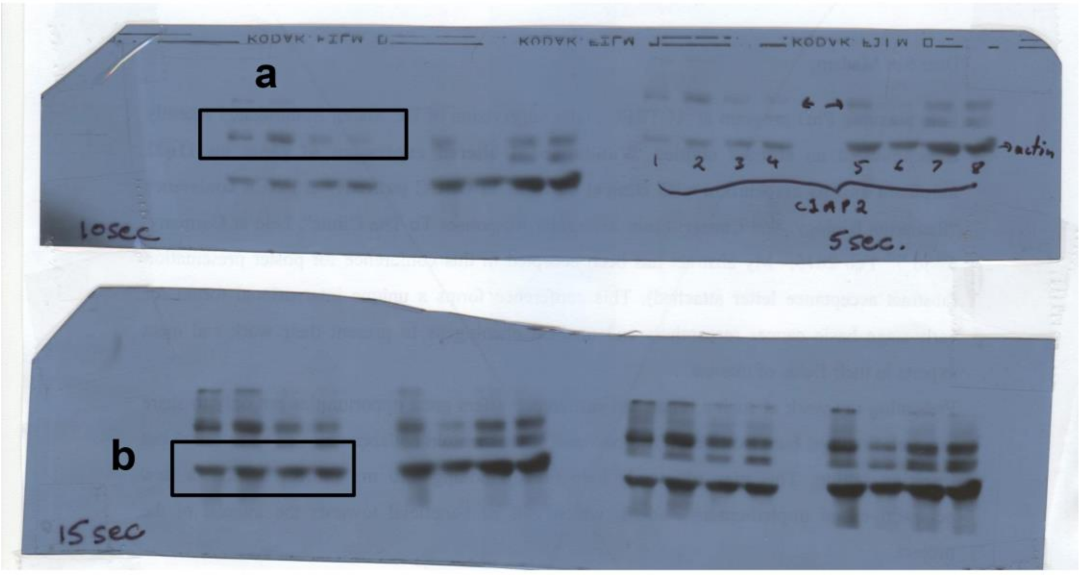
a
b
1 2 3 4
5 6 7 8
actin
cIAP2
10sec
5sec.
15 sec

S6-5

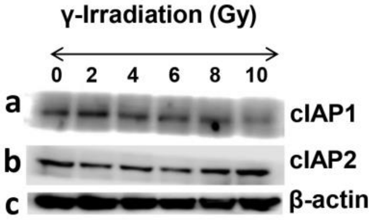
γ-Irradiation (Gy)
0 2 4 6 8 10
a
cIAP1
b
cIAP2
c
β-actin

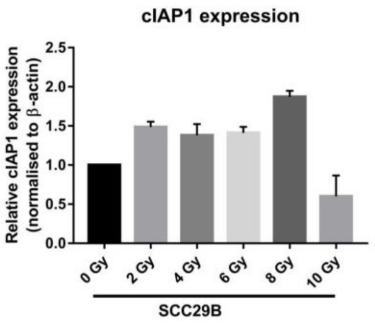
cIAP1 expression
Relative cIAP1 expression (normalised to β-actin)
2.5
2.0
1.5
1.0
0.5
0.0
0 Gy
2 Gy
4 Gy
6 Gy
8 Gy
10 Gy
SCC29B

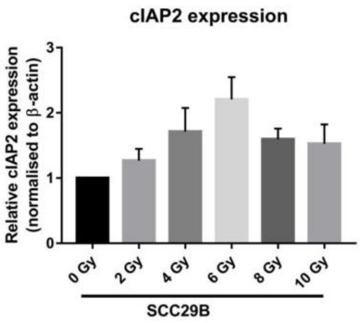
cIAP2 expression
Relative cIAP2 expression (normalised to β-actin)
3
2
1
0
0 Gy
2 Gy
4 Gy
6 Gy
8 Gy
10 Gy
SCC29B

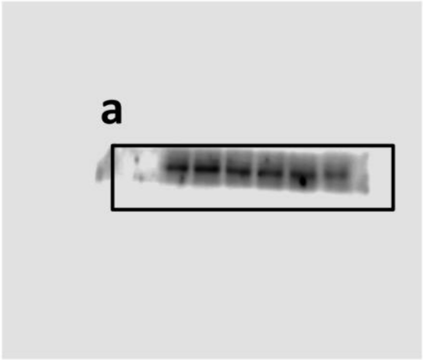
a

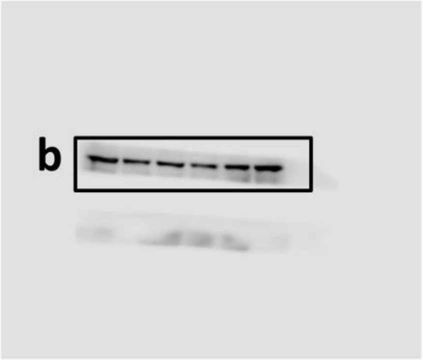
b

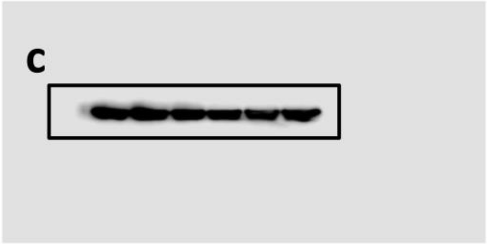
c

S6-6

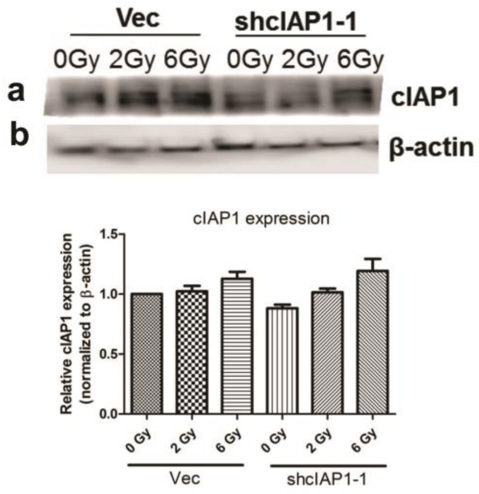
Vec
shcIAP1-1
0Gy 2Gy 6Gy 0Gy 2Gy 6Gy
a
cIAP1
b
β-actin
cIAP1 expression
Relative cIAP1 expression (normalized to β-actin)
1.5
1.0
0.5
0.0
0 Gy
2 Gy
6 Gy
0 Gy
2 Gy
6 Gy
Vec
shcIAP1-1

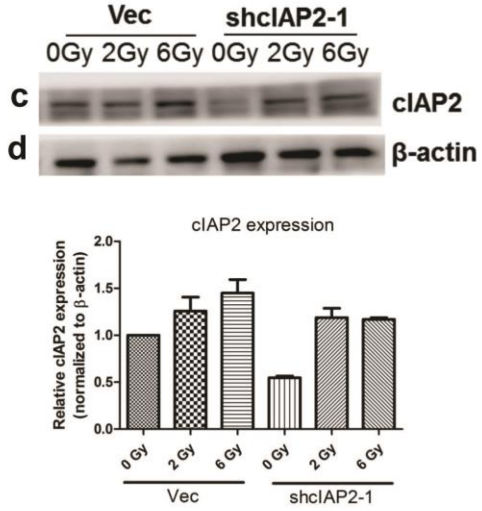
Vec
shcIAP2-1
0Gy 2Gy 6Gy 0Gy 2Gy 6Gy
c
cIAP2
d
β-actin
cIAP2 expression
Relative cIAP2 expression (normalized to β-actin)
2.0
1.5
1.0
0.5
0.0
0 Gy
2 Gy
6 Gy
0 Gy
2 Gy
6 Gy
Vec
shcIAP2-1

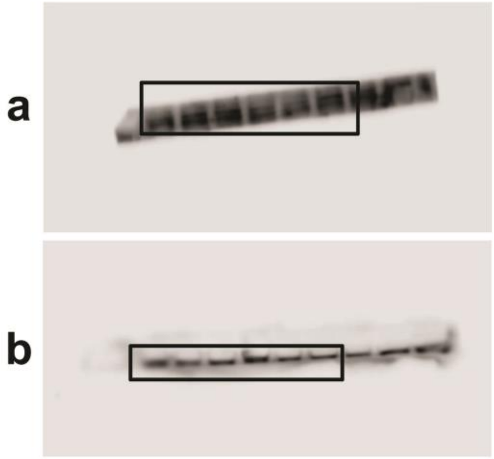
a
b

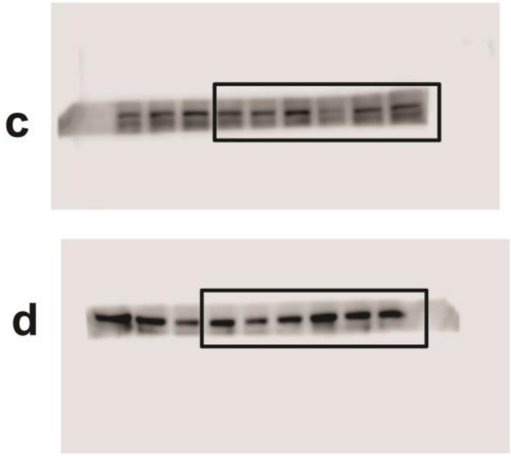
c
d
